# Medical Cannabis Increases Appetite but Not Body Weight in Patients with Inflammatory Bowel Diseases

**DOI:** 10.3390/nu16010078

**Published:** 2023-12-26

**Authors:** Naomi Fliss Isakov, Chen Seidenberg, David Meiri, Michal Yackobovitch-Gavan, Nitsan Maharshak, Ayal Hirsch

**Affiliations:** 1 Department of Health Promotion, School of Public Health, Faculty of Medicine, Tel Aviv University, Tel Aviv 6997801, Israel; 2 Department of Gastroenterology and Liver Diseases, Tel Aviv Medical Center, Faculty of Medicine, Tel Aviv University, Tel Aviv 6997801, Israel; nitsanm@tlvmc.gov.il (N.M.); ayalh@tlvmc.gov.il (A.H.); 3 School of Pharmacy, Hebrew University of Jerusalem, Jerusalem 9112002, Israel; chen.saidenberg@mail.huji.ac.il; 4 The Laboratory of Cancer Biology and Natural Drug Discovery, Faculty of Biology, Technion—Israel Institute of Technology, Haifa 3200003, Israel; dedimeiri1@gmail.com; 5 Department of Epidemiology and Preventive Medicine, School of Public Health, Faculty of Medicine, Tel Aviv University, Tel Aviv 6997801, Israel; michalyg2000@gmail.com

**Keywords:** medical cannabis, inflammatory bowel disease, appetite, nutritional status

## Abstract

We aimed to elucidate the effect of Medical Cannabis (MC) on appetite and nutritional status among patients with inflammatory bowel disease (IBD). A case series of patients with IBD were initiating treatment with MC for disease-related symptoms, at the IBD clinic of a tertiary referral medical center. Patients’ demographics, anthropometrics, medical history and treatment and MC use were systematically recorded. An appetite and food frequency questionnaire (SNAQ and FFQ) were filled before, and at 3 and 6 months of treatment. Patients with IBD initiating MC were enrolled (n = 149, age 39.0 ± 14.1 years, 42.3% female), and 33.6% (n = 50) were treated for improvement of nutritional status. A modest increase in appetite after 3 months was detected among all patients enrolled (Pv = 0.08), but there were no significant differences in energy or macronutrient intake, and in patients’ body mass index (BMI). A significant appetite improvement after 3 months was detected among 34.0% (n = 17) of patients, but this was not associated with increased caloric intake or BMI at 3 or 6 months. Among patients without increased appetite after 3 months of MC therapy, BMI decreased at 6 months (24.1 ± 3.7 vs. 23.4 ± 3.6, Pv = 0.010). MC may be a potential strategy to improve appetite among some patients with IBD, but not caloric intake or BMI.

## 1. Introduction

Inflammatory bowel disease (IBD), including Crohn’s disease, ulcerative colitis and pouchitis, are chronic inflammatory disorders of the gastrointestinal tract, which pose a major global public health burden [1,2]. Patients with IBD are at increased risk of poor nutrition and malnutrition, particularly patients with active inflammation and therapy refractory disease [3], although these conditions are also prevalent among patients with a quiescent disease [4]. Malnourished patients with IBD are more likely to be hospitalized, and among hospitalized patients malnutrition is an independent risk factor for venous thromboembolism, non-elective surgery, longer admission and increased mortality [5,6]. Therefore, screening for malnutrition risk, and managing undernutrition is recommended, using an appropriately trained multidisciplinary team of IBD experts [7]. 

Depending on the severity of the flares and the response to medication, IBD therapy includes anti-inflammatory agents, such as amino-salicylic acid (5-ASA), corticosteroids, immunosuppressants, biologic agents and advanced small molecules, such as Janus kinase inhibitors and sphingosine-1-phoshpate receptor modulators [8]. Despite multiple and novel therapeutic interventions, IBD treatment is still of limited efficacy and often accompanied by side effects, leading patients to seek alternative forms of treatment, such as medical cannabis (MC) [9]. In recent years, there is an increasing interest among patients and physicians, in the potential therapeutic role of MC for the treatment of IBD [10,11,12]. There are over 500 potentially active compounds within cannabis, with the two best studied compounds being cannabidiol (CBD) and Δ^9^-tetrahydrocannibinol (THC) [13,14]. It is postulated that phyto-cannabinoids modulate inflammation via the endocannabinoid system, with CBD and THC interacting with endocannabinoid receptors in the brain, enteric nervous system, gastrointestinal epithelial cells, and both macrophages and plasma cells [15,16,17].

There are only a few small studies evaluating the effect of cannabis or cannabinoids in active CD and UC, and to the best of our knowledge, there were no studies evaluating maintenance of remission in CD or UC. In a clinical trial assessing the effect of MC on moderately active CD, CBD was safe but had no beneficial effects on disease activity [18]. A more recent double-blind, randomized, placebo-controlled trial showed that CBD-rich cannabis treatment induced significant clinical and QOL improvement without significant changes on patient’s clinical disease activity, inflammatory parameters or endoscopic scores [19]. Similar results were demonstrated in observational studies. In a prospective follow-up of IBD patients treated with inhaled MC, patients showed improved quality of life, clinical disease activity index and weight gain after 3 months of therapy [20]. A case series from the UK Medical Cannabis Registry showed a significant increase in quality of life and decrease in anxiety after 3 months of therapy with MC [21]. Concluding from the evidence is limited due to different studies using different doses, formulations, and routes of administration of cannabis or cannabinoid, and the potential to use blinding during interventions. A meta-analysis on the topic concluded that evidence regarding the effects of cannabis and cannabidiol on CD and UC are uncertain [19]. Recently, another systematic review of the evidence established that, although cannabinoid usage in IBD treatment comes with promising results regarding clinical disease activity, quality of life and weight gain, high quality evidence has yet to detect the potential of different modes of administration and treatment dose [22].

MC is becoming a frequent treatment strategy for patients suffering from appetite loss, anorexia and cachexia, due to many different conditions, such as cancer, chronic pain, neurodegenerative disorders, etc. [23]. Around 40% of cancer patients use cannabis in places where access to MC as palliative care is legal, such as Canada, Germany, and Israel [24]. Despite the fact that MC and cannabis-based medicines (CBMs) are available in an increasing number of countries, the amount and quality of evidence for the use of these agents are low. In Israel, MC has been approved as a treatment strategy in patients with IBD for symptom control including decreased appetite and low dietary intake, abdominal or joint pain, low sleep quality, and management of anxiety and stress. Still, the evidence for MC induced appetite is scarce in patients with IBD. This study aimed to describe the effect of MC treatment on appetite and dietary intake among patients with IBD, in a prospective observational study of a MC specialized clinic for patients with IBD in a large tertiary medical center. 

## 2. Materials and Methods

This study was of an observational case series of patients, who were treated between October 2018 and April 2020 at the Tel Aviv Sourasky Medical Center (TASMC). The study protocol was approved by the TASMC Ethics Committee (TLV 0250-17, approved 16 July 2017, and TLV 0276-19, approved 10 July 2019) and all patients signed an informed consent form before enrollment.

### 2.1. Study Population

All patients who were prescribed to initiate treatment with MC for IBD-related symptoms at the IBD clinic were approached by the study team and asked to enroll in the study during their first visit, for cannabis prescription. 

Patients were prescribed MC according to their physician’s clinical judgment. Patients were not treated with MC in cases of pregnancy or lactation, psychiatric background, neurodegenerative disease and physical disability which may have increased falling risk among elderly and fragile patients, etc. Patients who were willing to participate were included if they had a medically stable disease, defined as no IBD related hospitalization or surgery within 6 months prior to enrollment, and no IBD treatment change during the 3 months prior to study enrollment. Exclusion criteria included: 18 > age > 80 years, unstable medical therapy, and inability to sign an informed consent or pregnancy. Patients were able to withdraw from the study at any time point, and were withdrawn if they had changed medical therapy, or in the case of pregnancy. To increase our study generalizability, no exclusion criteria were based on total energy intake. 

### 2.2. Data Collection

Upon enrollment, medical history was recorded, including demographics, medications, disease characteristics, extra-intestinal involvement and patient reported outcomes, (PROs) using standardized scales. The Harvey Bradshaw Index (HBI), the Simple Clinical Colitis Activity Index (SCCAI), and the Pouch Disease Activity Index (PDAI) were calculated for Crohn’s disease, ulcerative colitis and pouchitis, respectively. 

Anthropometrics were measured using a single standardized scale according to a unanimous protocol, and Body Mass Index (BMI) was recorded. Patients filled a standardized Food Frequency Questionnaire (FFQ) validated for the Israeli population and enabling assessment of long-term (3 months) dietary intake. From this, macronutrient and micronutrient intake was calculated for each patient at baseline. Patients were also requested to fill in the Hebrew translated Simplified Nutritional Appetite Questionnaire (SNAQ), to assess appetite level [25].

In addition, patients were questioned on history of past MC use, and recreational MC use, to classify as cannabis-naive or non-naive patients. MC treatment goals and prescription doses were documented by the study physician. Follow-up study visits were conducted at 3 and 6 months, and included a physician examination, anthropometric assessments, questionnaires, and documentation of MC treatment adherence, side effects and prescription changes. 

### 2.3. MC Treatment and Dose Calculation

An IBD specialist (AH), licensed for prescribing MC, prescribed treatment for alleviation of IBD-related symptoms with MC inflorescence, oil extraction or a combination. MC dose and composition were determined by the prescribing physician according to clinical characteristics and patients’ preferences. The prescriptions were fulfilled in authorized pharmacies supervised by the Israeli Ministry of Health. 

Prescription formulation (inflorescence, oil extraction or a combination), and dose (grams/month) of tetrahydrocannabinol (THC) to cannabidiol (CBD) were documented. For clarity and simplification, THC and CBD dose was calculated as grams (gr) × concentration. For example, a prescription of 20 gr of inflorescence with a concentration of 5% THC is presented as 1 gr/month of THC (20 × 0.05). Total prescription dose was calculated as the aggregated quantity (in grams) of THC and CBD. For example, a prescription of 20 gr of inflorescence with a concentration of 5% THC and 10% CBD is presented as 1 gr/month of THC (20 × 0.05) and 2 gr/month of CBD (20 × 0.1). A high dose of MC gr/month, THC gr/month, CBD gr/month and THC/CBD ratio was determined according to the sample dose median [MC dose > 21 gr/month, THC gr/month > 1.5 gr/month, CBD gr/month > 2.2 gr/month, THC/CBD ratio > 0.666]. 

### 2.4. Appetite, Nutritional Status and Intake

Nutritional status was assessed by using BMI. Underweight was defined as BMI < 18.5 kg/m^2^. 

Low appetite was defined according to the study sample fourth quartile as SNAQ appetite score ≤ 24 points. The difference in SNAQ appetite score between visits was calculated (SNAQ at 3 months–SNAQ at baseline). A significant increase in SNAQ score at 3 months was defined as a difference ≥ 3 points (the population third quartile).

Nutritional intake was evaluated from patients’ FFQs. Macronutrients intake and their proportional energy contribution were calculated. Total energy and protein intake were evaluated as kilocalories (kcal) and gr per body weight (kg), respectively. Macronutrient intake (protein, carbohydrate and fat) was evaluated as absolute values as grams (gr) or as percent of kcal. Micronutrient intake was also evaluated for assessment of intake of essential nutrients. 

### 2.5. Statistical Analysis

Continuous variables are presented as means ± SD and nominal variables as number and percentage. Pearson Chi-Square test was used to test the association between nominal variables. Comparison of continued variables between study groups was performed by the independent samples t-test for variables which distributed normally and by the Mann–Whitney test for variables with a skewed distribution. THC/CBD ratio was the only parameter that did not exhibit normal distribution. Normality was tested graphically and using the Shapiro Wilk test. Mixed-model repeated-measures analyses were conducted to evaluate the overtime trends in nutritional status and dietary intake (three visits). The mixed-model analysis enabled using all available data from the full cohort without imputing missing values. The models were specified with a within-group factor of time (baseline, 3 months and 6 months of follow-up), a between-group factor (according to treatment regimen) and the interaction of group with time. The data are expressed as estimated marginal means and standard errors. Statistical significance was set at *p* ≤ 0.05. All statistical analyses were performed using SPSS version 25.0 for Windows (SPSS Inc., Chicago, IL, USA).

## 3. Results

### 3.1. Study Population Characteristics

**Figure 1:**
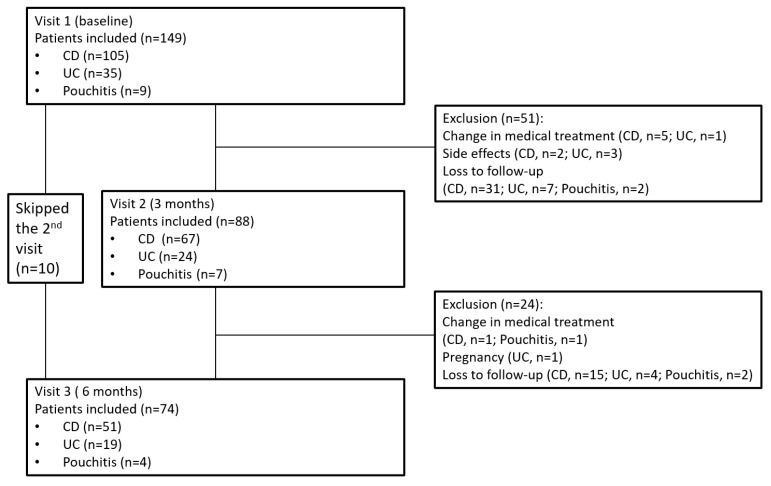
Flowchart of patient inclusion and follow-up. Abbreviations: CD—Crohn’s disease, UC—ulcerative colitis.

One hundred and forty-nine patients were recruited to the study (mean age 39.0 ± 14.1 years, 42.3% (n = 63) female, mean BMI 23.0 ± 3.9, 72.5% (n = 108) patients with CD). Of these, 88 patients were eligible for the analysis at 3 months, and 74 patients were evaluated at the 6 months visit (Figure 1). While clinical and anthropometric data were complete for all patients under follow-up, only a subsample of these patients filled in all questionnaires, including the SNAQ appetite and the FFQ questionnaires. Demographic and baseline clinical characteristics of the study population are depicted in Table 1.

### 3.2. Treatment Dose and Indications at Baseline

Patients were prescribed MC for various, non-exclusive, indications. 

The dose of THC prescribed was negatively correlated with patients’ age (r = −0.212, Pv = 0.041), and positively correlated with the simple clinical colitis activity index (SCCAI) score among patients with ulcerative colitis (UC) (r = 0.459, Pv = 0.016) and the clinical pouch disease activity index (cPDAI) score among patients with pouchitis (r = 0.742, Pv = 0.022). Altogether, patients with a clinically active disease (HBI > 4/SCCAI > 2/cPDAI > 2) were prescribed higher doses of MC compared to patients with a non-active disease (24.1 ± 7.6 gr/month vs. 19.6 ± 5.7 gr/month, Pv = 0.030), and were trending towards higher doses of THC (1.8 ± 1.3 gr/month vs. 1.3 ± 1.2 gr/month, Pv = 0.053), but not CBD. Fifty patients (33.6%) received MC for increasing appetite and improving nutritional status, and 99 (66.4%) for other indications, including control of joint pain, control of abdominal pain, improvement of sleep quality, and management of anxiety and stress. Comparison between patients prescribed MC for improving appetite vs. other indications is presented in Table 1. Patients who were treated with MC for improvement of nutritional status had lower BMI, and higher rates of unintentional weight loss, and were less biologically naïve compared to patients treated for other indications. 

The MC treatment indication for increasing appetite and dietary intake was associated with higher monthly dose of prescribed MC (25.7 ± 8.1 gr/month vs. 22.0 ± 6.8 gr/month, Pv = 0.005), and higher concentrations of THC (2.0 ± 1.5 gr/month vs. 1.5 ± 1.1 gr/month, Pv = 0.014), but not CBD. This was not seen for other indications, such as sleep quality (Pv = 0.109), control of joint pain (Pv = 0.102), or management of anxiety and stress (*p* = 0.214). 

Patients prescribed MC for improving appetite had significantly lower appetite score at baseline, compared to patients treated for other indications (24.5 ± 4.8 vs. 26.9 ± 4.4, Pv = 0.012). Surprisingly, mean energy and protein intake at baseline did not differ between these groups (33.3 ± 13.4 kcal/kg vs. 32.7 ± 11.9 kcal/kg, Pv = 0.879, and 1.6 ± 0.8 vs. 1.5 ± 0.6 gr/kg, Pv = 0.668, respectively). Similarly, macronutrient content and proportion of energy did not differ between the groups (for protein Pv = 0.688 and Pv = 0.605, for carbohydrates Pv = 0.628 and Pv = 0.863, and for fat Pv = 0.787 and Pv = 0.902). 

### 3.3. Effect of MC Treatment on Appetite and Dietary Intake

At three months of MC therapy, the mean SNAQ appetite score increased (Pv = 0.080), but there were no significant differences in energy or macronutrient intake, and in patients’ BMI (Table 2). In addition, there was no significant change in intake of sugar (% of kcal), saturated fatty acids, sodium, zinc, iron, vitamin C or essential amino acids.

Results did not change after stratifying according to adherence to therapy. The increase in appetite was most prominent in patients prescribed MC for improving appetite (24.9 ± 3.8 at baseline vs. 27.4 ± 3.7 after 3 months, Pv = 0.011). This was not seen in patients prescribed MC for other indications (26.2 ± 3.6 at baseline vs. 27.1 ± 4.2 after 3 months, Pv = 0.156) or in patients who changed or discontinued treatment regimen (24.5 ± 3.5 at baseline vs. 26.4 ± 3.6 after 3 months, Pv = 0.390). 

Interestingly, among patients with lower appetite scores, lower BMI and lower caloric intake, higher MC dose and high THC/CBD ratio dose were prescribed compared to patients with higher appetite scores. No significant change in appetite, dietary intake, or BMI was detected in either MC treatment regimens. Patients who were MC experienced reported higher appetite scores and higher caloric intake, but similar BMI, compared to MC naïve patients (Table 3).

**Figure 2:**
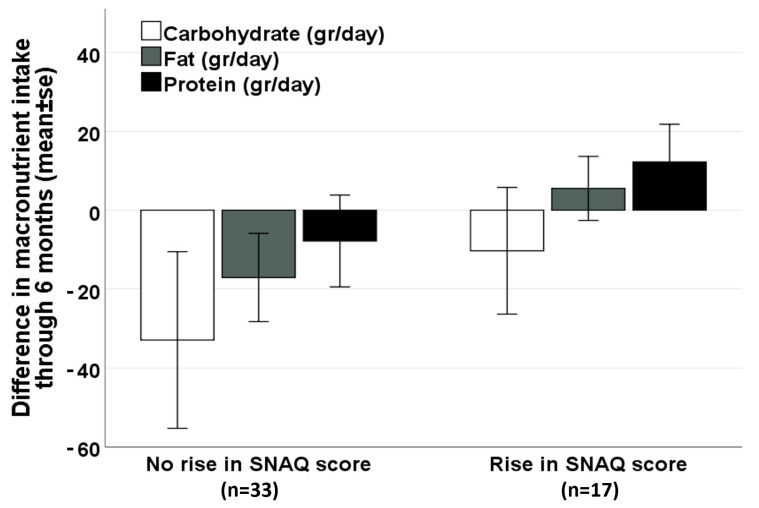
Difference in macronutrient intake after 6 months of MC therapy, by the change in appetite at 3 months (among patients treated for raising appetite and improving nutritional status, n = 50). Abbreviations: MC—Medical Cannabis, SNAQ—Simplified Nutritional Appetite Questionnaire.

On the other hand, among patients treated for raising appetite and improving nutritional status, following 3 months of MC therapy, 34.0% (17/50 patients) reported a significant increase in the SNAQ appetite score (≥3 points). These patients had a durable increase of SNAQ at 6 months (25.0 ± 3.9 at baseline vs. 28.0 ± 3.9 at 6 months, Pv = 0.009). This long-term increase in appetite was not associated with an increase in caloric intake (1953 ± 554 at baseline vs. 2019 ± 418 at 6 months, Pv = 0.718), nor in BMI (22.6 ± 3.1 kg/m^2^ at baseline vs. 22.9 ± 3.3 kg/m^2^ at 6 months, Pv = 0.507). However, among patients without a significant increase in appetite after 3 months of MC therapy, a significant decrease in BMI was noticed at 6 months (24.1 ± 3.7 at baseline vs. 23.4 ± 3.6 at 6 months, Pv = 0.010). A non-significant positive trend in fat and protein intake was seen among patients with a significant improvement in appetite, compared with a negative trend seen among patients without improved appetite (Figure 2).

Among the entire study group, four patients experienced a significant decrease in SNAQ appetite score after 3 months of MC therapy (8.0%), and another two patients reported this effect at 6 months of therapy (11.5%). 

### 3.4. Adherence to Treatment and Safety

At the second visit, 27 (30.6%) patients reported stopping the treatment or decreased their baseline MC prescribed dose. Side effects to treatment were reported among 62.5% (n = 55) of patients. The proportion of reported side effects did not differ between patients who changed or discontinued treatment compared to those who maintained their prescribed regimen throughout 3 months of follow-up (66.7% vs. 61.7% respectively, Pv = 0.665) (Supplementary Table S1). 

## 4. Discussion

In this study, we prospectively followed patients prescribed MC as part of their routine therapy at the IBD clinic of the TASMC, through 6 months of treatment. We found that, in these patients, MC was associated with a non-significant increase in appetite and BMI. Among patients treated for raising appetite and improving nutritional status, 34.0% (17/50 patients) reported a significant increase in appetite, following a positive trend in dietary intake.

More than 50% of the patients reported prior cannabis use for symptom control. This rate is in line with previous reports of cannabis use among patients with IBD, reported by 15–60% of patients in multiple surveys [26,27,28]. Patients treated with MC in our study for appetite improvement had lower appetite scores at enrollment, higher rates of pre-treatment weight loss, higher rates of underweight, and were more experienced with biologic therapy compared to patients treated for other indications. There was no difference from patients treated for other indications in disease duration, clinical disease activity, or extra-intestinal manifestations. Interestingly, both patient groups reported similar caloric intake and macronutrient distribution at baseline, implying a difference in either nutrient absorption capacity, basal energy requirements, or dietary quality. In accordance with previous studies, patients with IBD did not have increased energy expenditure as a direct result of their disease [5,29]. However, dietary intake of patients with IBD may be inadequate to meet even basic requirements, possibly due to malabsorption and disease-related or functional symptoms [30]. This may be aligned with the higher proportion of patients with an ileal pouch-anal anastomosis in the group of patients treated for raising their appetite, as these patients are characterized by high proportions of malabsorptive and functional symptoms [31].

Patients were treated with 23.2 ± 7.4 gr/month of MC, while MC dose and THC concentration was higher for patients who were treated to boost their appetite and improve nutritional status. This is compatible with reports supporting the effectiveness of high dose THC MC in increasing appetite, and a potential loss of appetite caused by a high dose CBD MC [32]. Indeed, following MC therapy, a third of the patients reported a significant increase in their appetite. On the other hand, increased appetite was not associated with significant differences in energy, macronutrient, and micronutrient intake or BMI. Eventually, after 6 months of MC therapy, a non-significant increase in protein and fat intake was noted, and a modest increase in BMI was detected among patients who reported an increase in their appetite after 3 months. Furthermore, patients whose appetite did not increase after 3 months of MC therapy, experienced a non-significant decrease in their nutrient intake, and a significant decrease in their mean BMI by 6 months. These results may imply that among some patients with IBD, MC treatment can be used to increase appetite, which may prevent weight loss. Notable is the fact that patients were prescribed MC without any other additional treatment per protocol, including dietary consultation, which may have had a significant impact on patient’s dietary choice and total intake. Consumption of MC was not associated with significant weight gain, perhaps implicating the need for a combined use of MC and dietary consultation, which may enhance clinical outcomes. There is also the possibility that cases with an active disease, malabsorptive disease, or those with functional symptoms, will not necessarily benefit from higher dietary intake. 

The stability in diet and in body weight is aligned with results seen in other reports of lower obesity rates among MC users [33], weight stability in MC users for other medical conditions [34] and in recreational users of MC [35,36]. Recent comprehensive reviews conclude that, although studies on the effects of MC on weight and metabolism have been performed, further investigation is warranted on a larger scale to help define the most appropriate dosage and effectiveness of cannabinoids in treating weight loss and cachexia [37,38]. Notably, the result of weight stability may be a preferable outcome in patients with various IBD symptoms often associated with weight loss, or some clinical circumstances in which patients have strict dietary needs, such as diabetes, some renal and liver disease and even celiac disease. The lack of weight gain may enhance use of MC for other indications in obese patients who may be reluctant to use MC due to the fear of gaining weight. 

Previous studies have shown conflicting results regarding the ability of MC to improve appetite, dietary intake and nutritional status. Some studies showed that cannabinoids had the potential to reduce pain, nausea, and vomiting, and improve appetite, caloric intake and body weight, in patients with cancer [39], multiple sclerosis [40], HIV [41], and anorexia nervosa [42]. On the other hand, multiple studies including meta-analyses reported low–moderate evidence to support the use of cannabinoids for the treatment of malnutrition [43,44]. Furthermore, some raised concern regarding side effects and potential harm to patients [43,45,46]. The mechanism of cannabinoid activity is related to G-protein coupled with cannabinoid 1 and 2 receptors (CB1/2). Cannabinoid receptors are expressed not only in the brain, but also in the gut and other peripheral organs involved in food intake, metabolism, and energy homeostasis. It is postulated that activation of the cannabinoid receptors by either endocannabinoids or exogenous cannabinoids acutely stimulates food craving, intake, and reward, whereas antagonism of the cannabinoid receptors reduces food intake and body weight [47,48]. However, more studies are required to shed light on the complexity of this cross-talk as, for example, acute versus chronic cannabis use may lead to different, and even opposite, outcomes [49]. In our study, a relatively large proportion of patients reported chronic use of cannabis, but we could not account for previous MC dose or duration of use. Patients who reported prior use of MC were expected to show moderate changes in diet, since they may have been treated with similar MC doses before and throughout the study, and indeed showed a non-significant decrease in caloric intake and BMI after 6 months of follow-up. These were not documented in patients who were naïve to MC. The large proportion of patients who were MC experienced may explain the negative results detected in our sample of patients. 

Limitations of our study include the observational nature of the study, including its intrinsic information bias. Nutritional status was assessed using BMI and might have been better represented using body composition analysis. Also, loss to follow-up and lack of compliance in answering questionnaires in long term follow-up visits are a major limitation but are anticipated in a real-world setting. Consequently, statistical power is limited and firm conclusions regarding each of the indications, clinical phenotypes or treatment strategies for the patients could not be performed due to the small number of patients in each group. Adherence to therapy, dietary and appetite endpoints were self-reported, potentially exposing the information to recall bias. Importantly, since MC was prescribed to all patients, and was not compared to another appetite-inducing strategies such as pharmacologic agents, we cannot conclude that it is more effective than any other treatment. Last, our study population is composed of patients treated in a tertiary hospital, limiting generalizability of results due to potential referral filter bias. On the other hand, advantages of this study include a real-world prospective design that enabled the obtaining of data in a meticulous and standardized manner using validated and detailed questionnaires. The study population was relatively large, with diverse demographic and medical characteristics, and treated for their disease in the same clinical setting, by the same physician, who clinically evaluated the patients throughout the study visits to prevent differential information bias. 

## 5. Conclusions

MC may be a potential strategy to increase appetite among some patients with IBD, which may prevent further weight loss, but is not associated with a significant increase in caloric intake or in BMI. Larger samples and longer follow-up studies are needed to produce high quality results with increased external validity, statistical power and long-term results. Additionally, future studies may gain by assessing the combined effect of MC and dietary consultation aimed at improving nutritional status in patients with IBD.

## Figures and Tables

**Table 1:** Demographic and clinical characteristics of the study population and a comparison between patients prescribed MC for improving appetite vs. other indications.

	Total Study Population (N = 149)	MC for Increasing Appetite/Improving Nutritional Status (n = 50)	MC for Other Indications (n = 99)	Pv
Demographic characteristics				
Age (years, mean ± sd)	39.0 ± 14.1	37.2 ± 14.4	40.0 ± 14.0	0.255
Gender–female %, (n)	42.3 (63)	44.0 (22)	41.4 (41)	0.763
Past smoking %, (n)	28.9 (43)	22.0 (11)	32.3 (32)	0.358
Current smoking %, (n)	26.2 (39)	26.0 (13)	26.3 (26)	
Previous Cannabis use %, (n)	37.6 (56)	42.0 (21)	35.4 (35)	0.541
BMI at baseline (kg/m^2^, mean ± sd)	23.0 ± 3.9	21.6 ± 3.8	23.8 ± 3.8	0.002
Underweight (BMI ≤ 18.5 kg/m^2^) %, (n)	10.1 (15)	18.0 (9)	6.1 (6)	0.022
Unintentional weight loss %, (n)	30.9 (46)	52.0 (26)	20.2 (20)	<0.001
Percent weight change 3 months prior to enrollment (%, mean ± sd) (n = 81)	−4.8 ± 15.5	−6.5 ± 15.9	−3.6 ± 15.3	0.409
IBD characteristics				
Disease type %, (n)				
CD	72.5 (108)	68.0 (34)	74.7 (74)	0.006
UC	21.5 (32)	20.0 (10)	22.2 (22)	
Pouchitis	6.0 (9)	12.0 (6)	3.0 (3)	
Disease duration (years, mean ± sd)	12.5 ± 10.9	12.5 ± 10.5	12.5 ± 11.2	0.996
Extra-intestinal manifestations %, (n)	60.4 (90)	66.0 (33)	57.6 (57)	0.609
Active clinical disease %, (n)	74.5 (111)	82.0 (41)	70.7 (70)	0.213
Biologic therapy experience %, (n)				
Naive	18.9 (25)	8.9 (4)	24.1 (21)	0.016
Past therapy	15.9 (21)	17.8 (8)	14.9 (13)	
Current therapy	65.7 (86)	73.4 (33)	60.9 (53)	
Prescribed cannabis treatment characteristics				
Treatment dose (gr/month) (mean ± sd)	23.2 ± 7.4	25.7 ± 8.1	22.0 ± 6.8	0.005
THC dose (gr/month) (mean ± sd)	1.7 ± 1.3	2.09 ± 1.5	1.5 ± 1.1	0.014
CBD dose (gr/month) (mean ± sd)	2.4 ± 0.9	2.4 ± 0.8	2.3 ± 0.9	0.495
THC/CBD dose ratio (median, range)	0.6 (0.03–5.0)	0.8 (0.03–5.0)	0.5 (0.03–5.0)	0.202

Abbreviations: MC—Medical Cannabis, BMI—Body Mass Index, CD—Crohn’s disease, UC—ulcerative colitis, IBD—inflammatory bowel disease, THC—tetrahydrocannabinol, CBD—cannabidiol.

**Table 2:** Nutritional outcomes of MC treatment throughout study visits.

	Baseline	3 Months	6 Months	Pv-Time
BMI (kg/m^2^, mean(se)) (n = 146)	23.1 (2.5)	22.7 (2.5)	22.9 (2.5)	0.825
SNAQ appetite score (mean (se)) (n = 101)	25.6 (0.4)	27.2 (0.6)	26.8 (0.5)	0.064
Total energy (kcal/day, mean (se)) (n = 60)	2193 (86)	2025 (91)	2068 (99)	0.627
Protein (% of kcal, mean (se)) (n = 60)	0.1 (0.006)	0.1 (0.006)	0.2 (0.007)	0.089
Carbohydrates (% of kcal, mean (se)) (n = 60)	0.4 (0.010)	0.4 (0.010)	0.4 (0.011)	0.808
Fat (% of kcal, mean (se)) (n = 60)	0.3 (0.007)	0.3 (0.007)	0.3 (0.008)	0.993

Mixed models repeated measures analysis. Values are presented as estimated means and standard errors, with post hoc least significant difference (LSD) pairwise comparisons. Abbreviations: MC—Medical Cannabis, BMI—Body Mass Index, SNAQ—Simplified Nutritional Appetite Questionnaire.

**Table 3:** Changes in appetite, dietary intake and BMI after 3 months and 6 months of MC treatment, stratified by treatment and cannabis use history.

SNAQ Appetite Score (Mean (se))							
		Baseline	3 Months	6 Months	Pv-Time	Pv-Treatment	Pv-Interaction
MC dose (gr/month)	High dose	23.1 (1.3)	25.1 (1.6)	24.5 (1.4)	0.297	0.026	0.950
	Low dose	25.6 (0.8)	28.2 (1.0)	26.3 (4.4)	0.604		
THC dose (gr/month)	High dose	24.5 (0.9)	26.3 (1.1)	26.3 (1.0)	0.367	0.371	0.581
	Low dose	23.8 (1.8)	26.6 (2.1)	23.8 (1.8)	0.425		
CBD dose (gr/month)	High dose	23.1 (1.0)	25.6 (1.2)	23.8 (1.1)	0.304	0.004	0.895
	Low dose	26.1 (1.1)	28.0 (1.5)	27.4 (1.1)	0.601		
THC/CBD dose ratio	High dose	25.0 (1.0)	26.9 (1.2)	26.8 (1.0)	0.391	0.050	0.633
	Low dose	23.4 (1.2)	25.8 (1.5)	23.4 (1.3)	0.422		
Prior cannabis use	MC Experienced	25.5 (0.9)	27.5 (1.2)	27.9 (1.0)	0.205	0.001	0.606
	MC naïve	22.0 (2.1)	23.4 (2.5)	19.6 (2.5)	0.574		
**Total energy (kcal/day, mean (se))**							
		**Baseline**	**3 months**	**6 months**	**Pv-time**	**Pv-treatment**	**Pv-interaction**
MC dose (gr/month)	High dose	2065 (509)	1531 (512)	2064 (516)	0.114	0.040	0.252
	Low dose	2331 (351)	2561 (376)	2213 (351)	0.759		
THC dose (gr/month)	High dose	2035 (580)	1649 (582)	1877 (580)	0.388	<0.001	0.381
	Low dose	2503 (378)	2897 (429)	2768 (429)	0.780		
CBD dose (gr/month)	High dose	2089 (236)	1861 (245)	2135 (279)	0.715	0.312	0.772
	Low dose	2349 (332)	2314 (367)	2158 (306)	0.904		
THC/CBD dose ratio	High dose	2202 (233)	1736 (240)	2048 (233)	0.376	0.082	0.268
	Low dose	2206 (344)	2711 (391)	2376 (391)	0.628		
Prior cannabis use	MC Experienced	2381 (264)	2301 (283)	2180 (264)	0.951	0.029	0.734
	MC naïve	1341 (221)	1048 (221)	1948 (221)	0.865		
**BMI (kg/m^2^, mean (se))**							
		**Baseline**	**3 months**	**6 months**	**Pv-time**	**Pv-treatment**	**Pv-interaction**
MC dose (gr/month)	High dose	20.4 (0.4)	20.5 (0.5)	20.3 (0.5)	0.755	<0.001	0.857
	Low dose	22.1 (0.5)	22.6 (0.6)	22.5 (0.6)	0.981		
THC dose (gr/month)	High dose	21.1 (2.0)	21.3 (2.1)	21.5 (2.1)	0.833	0.548	0.867
	Low dose	21.5 (1.0)	22.0 (1.1)	21.5 (1.1)	0.870		
CBD dose (gr/month)	High dose	20.7 (2.1)	21.5 (2.1)	21.1 (2.1)	0.988	0.139	0.819
	Low dose	21.9 (0.5)	21.8 (0.7)	21.9 (0.6)	0.679		
THC/CBD dose ratio	High dose	21.4 (0.5)	21.5 (0.6)	21.6 (0.6)	0.790	0.830	0.933
	Low dose	21.1 (0.5)	21.7 (0.6)	21.4 (0.6)	0.959		
Prior cannabis use	MC Experienced	21.1 (0.5)	21.7 (0.7)	21.3 (0.6)	0.975	0.922	0.970
	MC naïve	21.9 (0.8)	21.7 (1.0)	21.6 (1.0)	0.812		

Mixed models repeated measures analysis. Values are presented as estimated means and standard errors. Abbreviations: MC—Medical Cannabis, BMI—Body Mass Index, SNAQ—Simplified Nutritional Appetite Questionnaire, THC—tetrahydrocannabinol, CBD—cannabidiol.

## Data Availability

Data is unavailable due to privacy restrictions.

## Supplementary Materials

The following supporting information can be downloaded at: https://www.mdpi.com/article/10.3390/nu16010078/s1. Table S1: Comparison of patients according to their compliance with MC therapy.
