# Supplementary material for: Medical Cannabis Increases Appetite but Not Body Weight in Patients with Inflammatory Bowel Diseases
Source: Nutrients. 2023 Dec 26;16(1):78. doi: 10.3390/nu16010078 (PMC10781068; doi:10.3390/nu16010078)
Supplement: Supplementary file 1 [file nutrients-16-00078-s001.zip › nutrients-2758296-supplementary.pdf]

Table S1. Comparison of patients according their compliance to MC therapy.

	Total population (n=88)	High compliance (n=61)	Low compliance [a] (n=27)	P
Treatment indications %, (n)				
Increase appetite and dietary intake	42.5 (37)	39.3 (24)	48.1 (13)	0.477
Other indications	57.9 (51)	60.6 (37)	51.9 (14)	
Prescribed cannabis treatment characteristics				
Treatment dose (gr/month) (mean±	23.1±7.5	22.4±6.9	25.0±7.0	0.276
THC dose (gr/month) (mean±sd)	1.7±1.2	1.6±1.1	1.5±0.7	0.771
CBD dose (gr/month) (mean±sd)	2.4±0.9	2.4±0.9	2.5±0.6	0.639
THC/CBD dose ratio (median, rang	0.6 (0.03-5.0)	0.6 (0.03-5.0)	0.7 (0.2-1.25)	0.821
Adverse events %, (n)				
Dizziness	3.4 (3)	1.7 (1)	7.4 (2)	0.175
Fatigue	21.8 (19)	23.3 (14)	18.5 (5)	0.615
Confusion	1.1 (1)	1.7 (1)	0.0 (0)	0.500
Mood lability	16.1 (14)	15.0 (9)	18.5 (5)	0.679
Palpitations	5.7 (5)	8.3 (5)	0.0 (0)	0.122
Dry mouth	23.0 (20)	26.7 (16)	14.8 (4)	0.224
Increased appetite	28.7 (25)	28.3 (17)	29.6 (8)	0.902
Weight gain	10.3 (9)	8.3 (5)	14.8 (4)	0.358
Previous Cannabis use %, (n)	40.9, (36)	40.9, (25)	29.6, (8)	0.150

[a] Low compliance to MC therapy was defined as a decrease or discontinuation of MC treatment, according to the patient's own decision; patients with high compliance were those who maintained their prescribed regimen throughout 3 months of follow-up
